# Promoting computational thinking through project-based learning

**DOI:** 10.1186/s43031-021-00033-y

**Published:** 2021-08-02

**Authors:** Namsoo Shin, Jonathan Bowers, Joseph Krajcik, Daniel Damelin

**Affiliations:** 1 CREATE for STEM Institute, Michigan State University, 620 Farm Lane, Suite 115, East Lansing, MI 48824 USA; 2 The Concord Consortium, 25 Love Lane, Concord, MA 01742 USA

**Keywords:** Project-based learning, Computational thinking, Computational modeling, Science, High school

## Abstract

This paper introduces project-based learning (PBL) features for developing technological, curricular, and pedagogical supports to engage students in computational thinking (CT) through modeling. CT is recognized as the collection of approaches that  involve people in computational problem solving. CT supports students in deconstructing and reformulating a phenomenon such that it can be resolved using an information-processing agent (human or machine) to reach a scientifically appropriate explanation of a phenomenon. PBL allows students to learn by doing, to apply ideas, figure out how phenomena occur and solve challenging, compelling and complex problems. In doing so, students  take part in authentic science practices similar to those of professionals in science or engineering, such as computational thinking. This paper includes 1) CT and its associated aspects, 2) The foundation of PBL, 3) PBL design features to support CT through modeling, and 4) a curriculum example and associated student models to illustrate how particular design features can be used for developing high school physical science materials, such as an evaporative cooling unit to promote the teaching and learning of CT.

## Introduction

To solve complicated everyday problems, it is important for students to apply^1^big ideas of science and science practices so that they can make well grounded decisions while also considering multiple perspectives on both a local and global scale. Modern scientific exploration relies heavily on computational modeling and analysis, which requires that students use computational thinking to understand phenomena. Computational thinking (CT) is recognized as the thought processes needed to support problem solving, which involves decomposing a problem, creating modifiable artifacts (e.g., computational models) based on data generation, and revising those artifacts through testing and debugging and iterative refinements (Damelin, Stephens, & Shin, 2019; Wing, 2006). Computational modeling refers to building models that can be simulated using computers to predict outputs instantaneously based on novel data added to the model. CT, particularly testing and debugging through simulations and data generation, is necessary to refine the computational model continuously as new data becomes available to reach scientifically appropriate explanations of a phenomenon.

**Fig. 1:**
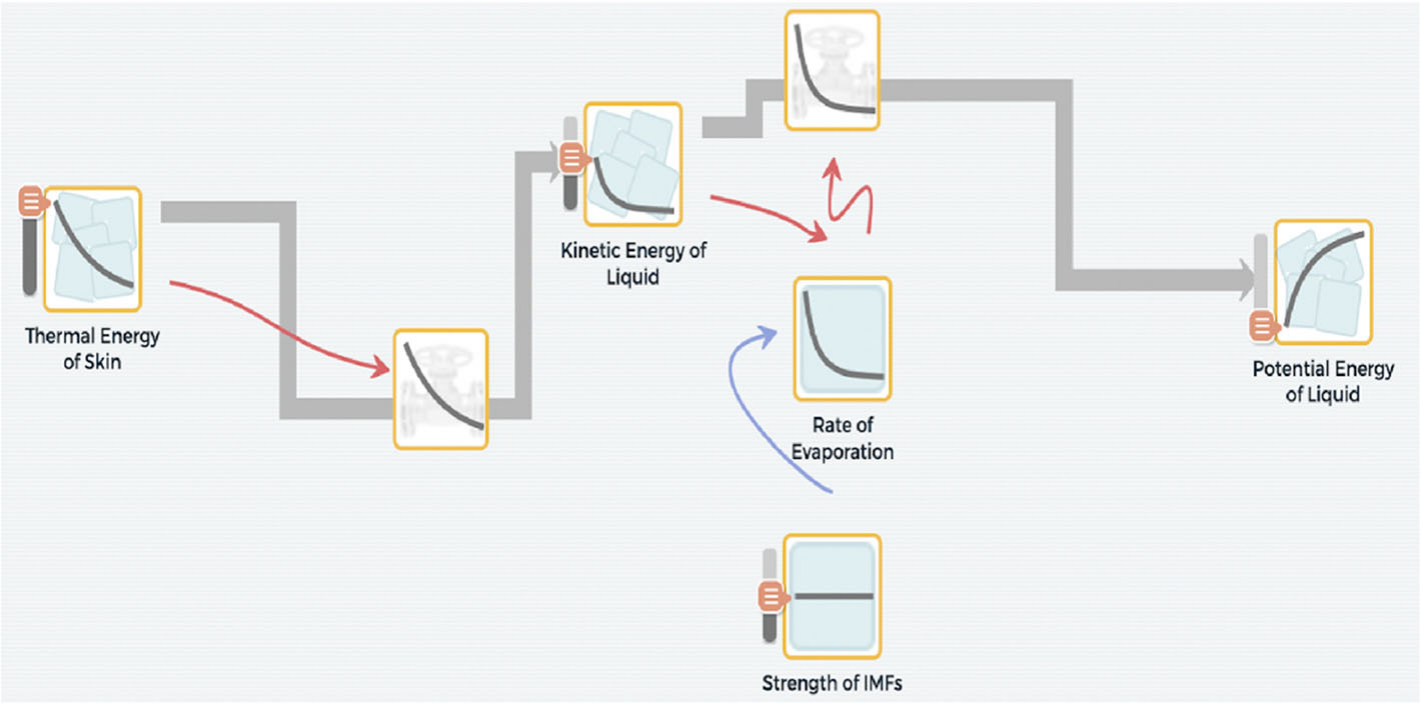
Ideal model of evaporative cooling phenomenon. Note: This model of evaporative cooling focuses on how thermal energy from the skin (thermal energy of skin) is used to warm up the liquid (kinetic energy of liquid) and ultimately drive the process of evaporation (rate of evaporation), resulting in an increase in potential energy of that substance (potential energy of liquid in the form of a gas)

We propose that the principles of project-based learning (PBL) provide opportunities for students to participate in CT. PBL focuses on learning by doing (active constructions) by using science ideas and practices where students can create computational models collaboratively through defining a phenomenon, and then, testing, debugging, and refining their own scientific understandings about the relationships and processes of phenomena they notice in the world (Krajcik & Shin, in press). The goal of this paper is to explore how PBL can support students in engaging with CT through computational modeling. This paper includes a description of 1) CT and its associated aspects, 2) the foundations of PBL, 3) PBL design features to support CT through modeling, and 4) a curriculum example and associated student models to present how the design features can be used for developing high school science materials, specifically an evaporative cooling unit for promoting the teaching and learning of CT. The system that students are modeling in this unit focuses on predicting how the evaporation of a liquid off of human skin causes a person to feel colder as the thermal energy of their skin is transferred to the potential energy of the evaporating molecules. This particular system benefits from a computational approach to modeling as it allows for students to explore computational aspects of this phenomenon such as how the rate at which a liquid evaporates off of the skin decreases overtime as the person loses more thermal energy (See Fig. 1 for the ideal model of the evaporative cooling unit).

## Theoretical background

### Computational thinking

Computational thinking (CT) is not only useful for computer scientists and software engineers, but is beneficial for everyone trying to understand phenomena, solve complex problems, or analyze multiple possible outcomes to make an informed decision (Grover & Pea, 2017; Wing, 2006). CT can frequently be used to gain insight into phenomena and find a computational solution, which is a computational model in our context. We define “computational solutions” or “computational models” as algorithmically defined artifacts (products) that can be modified using novel data to allow us to see a range of results and potential solutions. For example, during the Covid-19 crisis, scientists developed and regularly revised computational models that predict the spread of the virus. These computational models can be simulated and the outputs of the simulation are in turn interpreted by individuals to inform their behavioral responses to the pandemic. CT based on the simulation modeling perspective centers on five aspects: (CT1) decomposing a problem such that it is computationally solvable, (CT2) creating computational artifacts through algorithmic thinking, (CT3) generating, organizing, and interpreting data, (CT4) testing and debugging, and (CT5) making iterative refinements.

#### CT1

*Decomposing a problem such that it is computationally solvable* involves breaking down a problem into identifiable elements and logically categorizing them into essential and irrelevant elements. It makes a problem more tractable, the problem-solving process more manageable, and therefore easier to find a computational solution. Students begin by recognizing the elements of a phenomenon; they then select specific elements that are essential for understanding the phenomenon, while discarding irrelevant elements. As students decompose a phenomenon they also describe a clear goal and specify questions that need to be answered as well as an approach to answering these questions.

#### CT2

*Creating computational artifacts through algorithmic thinking* refers to crafting viable computational models via iterative revision processes such that the model can explain, simulate, or predict phenomena. As students construct computational models, they encode relevant variables and relationships among variables in a way that computers can interpret them (e.g., in our curriculum, simulation). Algorithmic thinking is grounded in developing a logical set of operations for manipulating an artifact, and requires students to continuously revise this artifact, allowing them to produce a computational model as a result of those manipulations.

#### CT3

*Generating, organizing, and interpreting data* involves identifying distinctive correlations and patterns from datasets to make sense of phenomena (Schwarz, Passmore, & Reiser, 2017; Weintrop et al., 2016). During this process, mathematical relationships between variables are represented visually to show the patterns or trends of the data. It is essential to find meaningful patterns and correlations in data in order to make claims grounded in evidence obtained from analyzing data and to communicate findings to others. The output of this data generation provides evidence for students to test their models against, thereby facilitating *debugging of* their computational models*.*

#### CT4

**Fig. 2:**
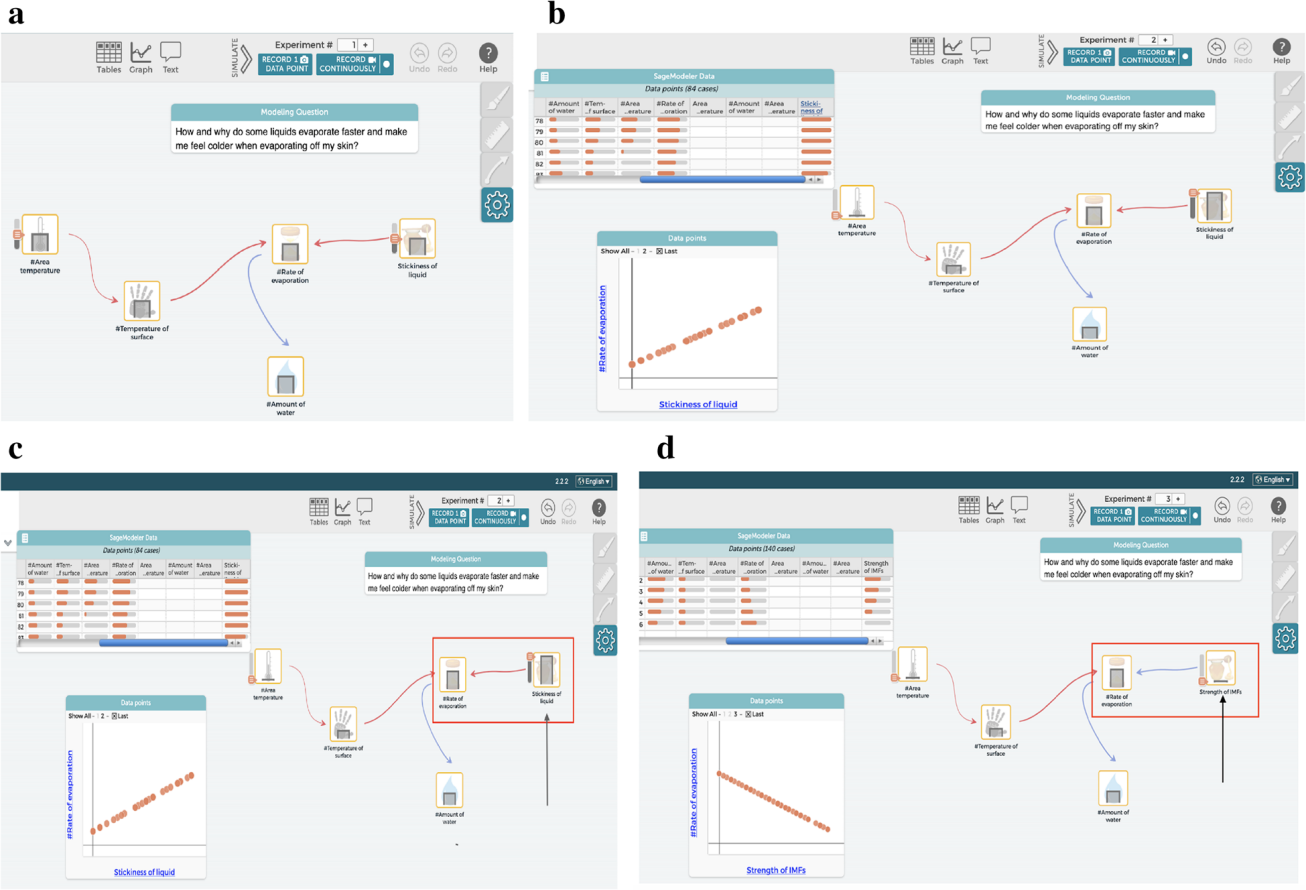
Iterative revisions of students’ models through engaging in CT: testing and debugging, data generation, and iterative refinement

*Testing and debugging* involves detecting issues in a solution that doesn’t match the phenomena or real world data, fixing these issues based on the behavior of the artifact, and confirming the solution using multiple starting conditions. Logical thinking is necessary for manipulating the initial value of a model, and for investigating the impact of these changes on the outputs (Fig. 2b for manipulating initial input using the “slider bars” of each element after simulating a model; Fig. 2c for identifying the issues in a model and debugging those issues; and Fig. 2d for retesting the model). Additionally, testing and debugging refers to fixing incorrect definitions of the problem or phenomena, incorrect conceptual understandings of the phenomena, or incorrect representations of one’s conceptual understanding of the phenomenon.

#### CT5

*Making iterative refinements means* evaluating the appropriateness of a solution (e.g., from a computational model) based on the explanatory goal as well as the available supporting evidence. It involves comparing the output obtained from simulating a computational model with experimental or real-world data, or expected outcomes to refine the model and analyze whether a model behaves as expected (Grover & Pea, 2017; Weintrop et al., 2016). In addition, making iterative refinements involves making periodic modifications to account for new evidence or new insights to optimize a model that allows for predicting the behavior of a wide range of phenomena with similar structures.

### Project-based learning

Project-based learning involves students experiencing and making sense of phenomena as they take part in various science practices with big ideas of science through constructing tangible products in collaborative activities (Krajcik & Shin, in press). Key learning principles of PBL include 1) active construction of tangible products, 2) using meaningful questions to engage with various practices and big ideas in compelling real world contexts, 3) active collaboration, and 4) use of cognitive tools. First, the active construction of tangible products – external representations that result from knowledge building (e.g., computational models) – supports students as active learners to develop their knowledge through doing (Miller & Krajcik, 2019; National Academies of Sciences, Engineering, and Medicine, 2018). The notion of active construction is grounded in the learning theory that students learn effectively when they construct and reconstruct what they learn from new experiences through integrating with their prior knowledge (Harel & Papert, 1990; National Academies of Sciences, Engineering, and Medicine, 2018; National Research Council, 2007 & 2012; Papert, 2005; Smith, Wiser, Anderson, & Krajcik, 2006). Since the development of deep understanding is a continuous, developmental process and takes time, students need to engage in student-centered learning experiences for a sustained period of time (National Research Council, 2012). In PBL, students construct tangible products over the course of inquiry activities that provide opportunities to connect ideas together for making sense of phenomena that are important to them.

Secondly, while students actively construct their products, they participate in meaningful tasks dealing with compelling problems related to their life and mirroring the practices in the field of science and engineering (Krajcik & Shin, in press; National Research Council, 2012; National Academies of Sciences, Engineering, and Medicine, 2018). For example, while students investigate a phenomenon through modeling to answer questions that require computational thinking (e.g, how does the amount of ice affect the change in temperature of the Earth?), they engage in computational thinking intersecting with other practices including developing models, analyzing and interpreting data, explaining and predicting phenomena, and designing solutions to create scientifically appropriate computational models. The underlying premise of PBL is that ideas and practices must be learned in a harmonious manner for students to see the value of the learning activities they perform (Brown, Collins, & Duguid, 1989).

Lastly, to scaffold the construction of knowledge products using various practices, PBL employs research findings on the important roles of collaboration (Brown & Campione, 1994) and cognitive tools (Chiu et al., 2018; Krajcik & Mun, 2014; Tinker, 1997) in learning. PBL activities are centered in sharing, using, and debating ideas with others to help students in making sense of phenomena by using their prior knowledge (Blumenfeld, Marx, Krajcik, & Soloway, 1996; Hasni et al., 2016). Cognitive tools are used to facilitate collaborative activities for obtaining, collecting, and sharing ideas and information, and creating products, such as computational models. Collaborative cognitive tools using enhanced technology expand the range and type of phenomena that students can explore and experience beyond their natural ability. They make it possible to collect and analyze data with technological instruments (e.g., probes, data and graph generation tools), feel unseen phenomena through advanced technologies (e.g, augmented and virtual reality), communicate virtually over long distances using digital methods (e.g, website, collaboration editing tools), and create sophisticated products using a computer (e.g., computational modeling tools).

Such PBL environments that have emerged from these aforementioned learning principles – active construction of tangible products, experiences of science practices with meaningful tasks driven by compelling, computationally solvable questions, collaboration, and use of cognitive tools – provide opportunities for students to engage in computational thinking to explore, explain, and predict phenomena. For example, teachers or curriculum developers can design learning activities based on PBL for students to create computational models as a tangible product using science practices integrated with key science ideas or concepts to explore a phenomenon or solve a compelling problem driven by a CT required meaningful question. Students construct and revise these models in collaborative learning environments using various cognitive tools. In our work in high school science classrooms, this often occurs as students participate in CT using a computational modeling tool called SageModeler (https://sagemodeler.concord.org).

## PBL design features for computational thinking through modeling

The theoretical foundations of PBL and CT guide us to develop PBL design features for supporting students’ CT in the context of modeling. These PBL design features include:

1. Focusing on *learning goals* such that students are able to demonstrate mastery of both science ideas and CT practice.
2. Starting with a *driving question grounded in CT* that students find meaningful to sustain engagement and drive learning through CT.
3. Exploring the driving question *by participating in science practices that intersect with CT* (e.g., asking questions, developing and using models, planning and carrying out investigation, analyzing and interpreting data, constructing an explanation and designing a solution) so that students see the value of and transfer their learning to everyday situations.
4. C*reating a set of tangible CT products in collaborative sensemaking activities* to address the driving question so that students construct their own knowledge as active learners.
5. *Scaffolding with learning technologies* (e.g., computational modeling tools, discourse tools) to help students participate in activities and engage in CT beyond their normal abilities.

### Design feature 1. Focusing learning goals

This feature ensures that learning materials support students in engaging with CT and achieving a deep understanding of science ideas and practices. These learning goals guide curriculum developers in providing sufficient information and materials for students to explore a phenomenon, define the elements of the phenomenon, and map their relationships to specify the boundaries of the phenomenon through computational modeling. Our learning goals are performance-based learning goals that integrate big ideas of science with science practices to reflect the professional disciplinary practices of working scientists (Krajcik, McNeill, & Reiser, 2008).

To specify what aspects of CT we expect students to engage with alongside appropriate big ideas in the evaporative cooling curriculum, we selected a national standard (High School Physical Sciences 3–2) – “Develop and use models to illustrate that energy at the macroscopic scale can be accounted for as a combination of energy associated with the motions of particles (objects) and energy associated with the relative positions of particles (objects). ”– to guide overall curriculum development (NGSS Lead States, 2013). We then unpacked the big ideas of this standard to break it down into several more manageable learning goals, such as “ask questions about observations of the evaporation of liquids to describe how energy is being transferred from the heat of the skin to the potential energy of the gas molecules” and “develop computational models to explain how energy transfer affects matter changes during evaporation” to shape the course of the curriculum with a smaller scope of lessons. Since these learning goals integrate key science ideas and CT related science practices, they can be used as guides for designing and aligning the learning tasks (e.g., investigations, modeling activities, and artifacts creations) and assessments to support CT at both the curriculum and individual lesson level in a coherent manner.

### Design feature 2. Starting with a driving question

A driving question refers to a question grounded in an anchoring phenomenon that students need to answer through subsequent learning activities and through the construction of their computational models. A driving question has important roles for organizing the sequence of learning activities, providing a context for student engagement in both science content and practices, and guiding continuity and coherence to the full range of activities (Krajcik & Shin, in press). This feature supports students in the *problem decomposition* aspect of CT by describing a goal for their tasks and specifying a question that needs to be answered through investigative activities; this feature also supports *problem decomposition* by providing opportunities to explore elements related to a phenomenon (Bielik, Damelin, & Krajcik, 2019).

To fully appreciate a driving question grounded in CT, students must first experience the phenomenon underlying the CT grounded driving question in an authentic manner. Our curricula start with students experiencing a phenomenon that fosters curiosity while also providing an authentic need for CT. The goal of this initial experience with the phenomenon is to provide students with rich contexts so they can explore the various elements that are present in the phenomenon. For example, in our “evaporative cooling” unit students begin by participating in an activity where they place various contact safe liquids on their skin (water, acetone, rubbing alcohol, and cooking oil) and observe the rate of evaporation of these substances and if these substance make their skin feel cooler as they evaporate. As students work with the phenomenon of evaporative cooling, students must be able to participate in the CT aspect of *decomposing a phenomenon* so that they can identify the relevant elements of the system that they will need to include in their computational models. Many of these elements, such as the “rate of evaporation” and the “amount of heat on my skin” are computational in nature and have interesting behavioral patterns (that can be modeled algorithmically) thereby creating an authentic need for students to apply CT to address the questions that they will have about this phenomenon.

**Fig. 3:**
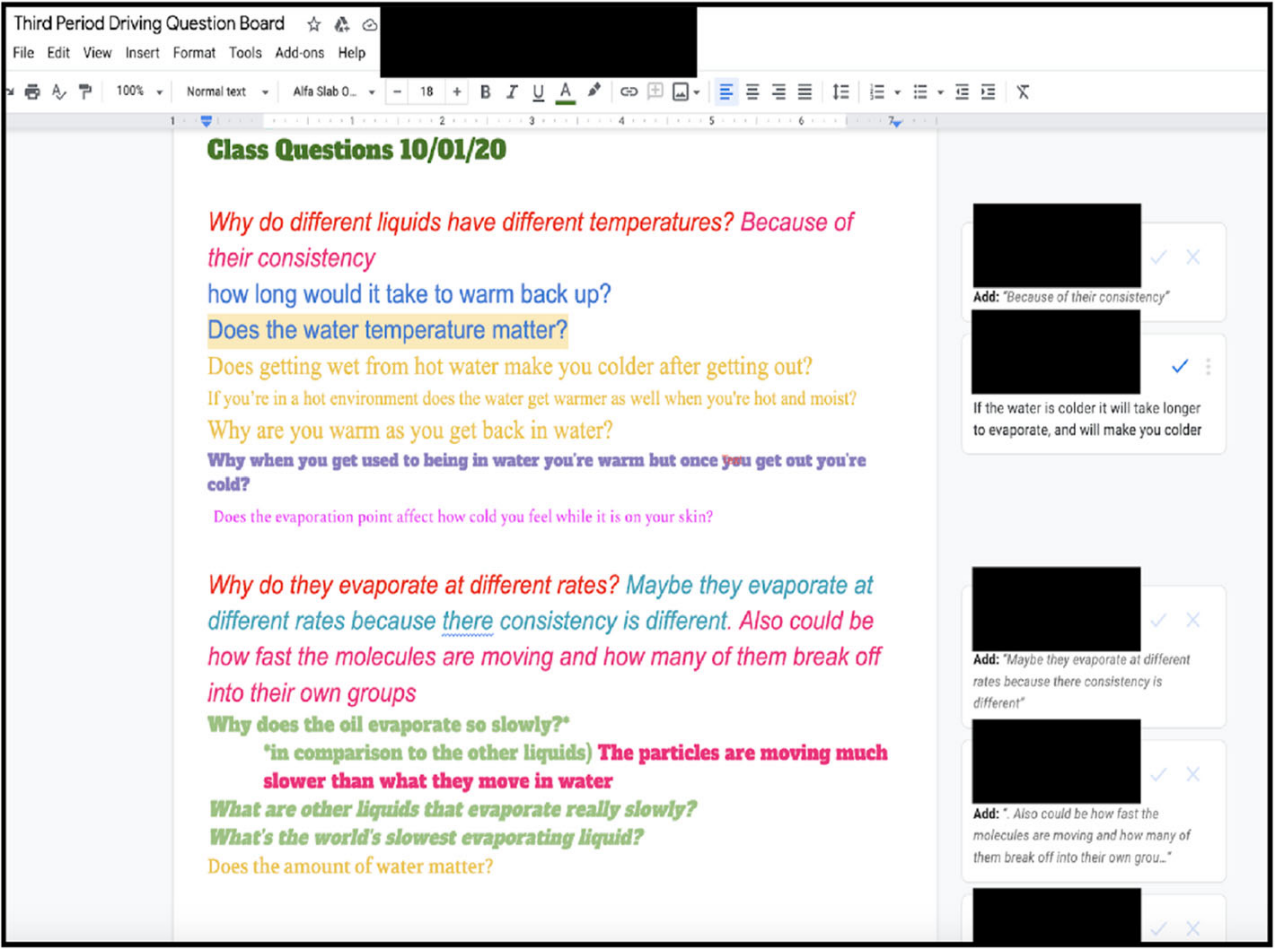
Driving Question Board using a google doc

Once students have explored the principle phenomenon, they investigate the driving question and/or the questions they generated with their partners. In the “evaporative cooling” unit, we gave the students the following driving question: “Why do I feel colder when I am wet than when I am dry?” To respond to the driving question, students need to break down the phenomenon into more specific elements that better suit the aims of the driving question and further investigation. To accomplish this, students brainstorm more specific sub questions and organize these questions on a Driving Question Board (DQB) that serves as a cognitive tool that would address specific components of the driving question and help scaffold student discourse during subsequent investigations within this unit (see Fig. 3).

For example, in Fig. 3, students wrote down the sub-question “*how long would it take to warm back up?*” This particular sub-question is computational in nature as it requires students to consider the dynamic nature of this system (how its behavior will change over time) as well as the various components that influence the behavior of this system. Additional sub-questions, such as “why do they (the liquids) evaporate at different rates?” and “why does the oil evaporate so slowly?” are also computational in nature and help students develop an authentic need to engage with CT as they address the initial driving question of “Why do I feel colder when I am wet than when I am dry?”

Using the DQB helps students narrow the scope of the phenomenon by characterizing the elements in the phenomenon and can help them plan an experimental approach to collect appropriate data to use as evidence. Throughout subsequent lessons we provide a wide range of supplementary phenomena (such as an experiment involving measuring the change in temperature as ice melts into liquid water and then boils) that relate to lesson specific learning goals and correspond to student generated questions on the DQB. After engaging in these supplementary phenomena, students return to the DQB to see if their new observations can be used to address one or more of the questions they wrote on the DQB as well as how this new information pertains to the overarching driving question “Why do I feel colder when I am wet than when I am dry?” By embedding multiple compelling phenomena into the unit, we support varied learning experiences that encourage students to capture essential elements across phenomena and transfer their understanding of one phenomenon to explain other phenomena and address the driving question.

**Fig. 4:**
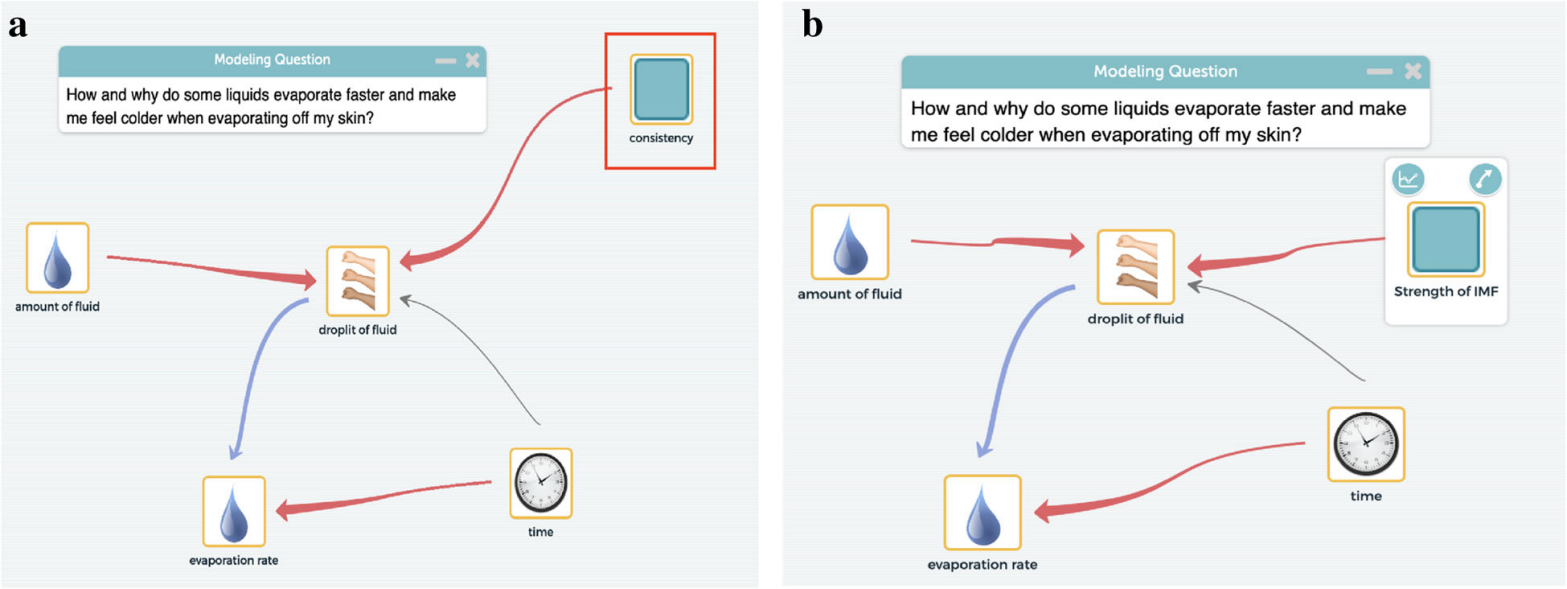
Engagement of CT problem decomposition through activities of discussing DQ and observing various phenomena (recreated based on classroom discourse). Note. DQ. Driving question. **a** Element present in initial model. Note: Elements in an initial model by observing an anchoring phenomenon, discussing DQ, and developing paper-pencil models. **b** Element present in revised model. Note: Select key elements by deleting irrelevant ones and adding new ones through experiencing various phenomena through new investigation and revisiting DQ

Periodically returning to the DQB after completing investigative activities helps students iteratively refine their models by adding or deleting elements in their computational model as shown in Fig. 4. In this particular example, the students added the element of “strength of intermolecular forces (IMFs)” to their models after completing an investigation that managed to address the student generated question of “why do different liquids have different temperatures?” By connecting the results of this investigation on IMFs back to the student generated question as well as the previous answers of other students “because of their consistency” the students were able to connect the macro-level phenomenon of “consistency” (or viscosity) with the newly introduced molecular concept of IMFs. As shown by the models below, the students removed “consistency” as an element and added “strength of IMF” to their model.

### Design feature 3. Participating in science practices

This feature engages students in science practices that intersect with CT by exploring driving questions through subsequent investigations over an extended time period. Our PBL materials provide specific instructional supports on how to scaffold students in various CT aspects. In particular we have focused on how to support students in constructing and revising models in a way that also facilitates CT. We define five modeling processes for engaging students in the iterative model developments as well as CT including: M1) characterize problem or phenomenon to model, M2) define the boundaries of the system, M3) design and construct model structure, M4) test, evaluate, and debug model behavior, and M5) use model to explain and predict behavior of phenomenon or design solution to a problem (Eiden et al., 2020). These modeling practices are grounded in different aspects of CT (Damelin et al., 2019). We developed activities to engage specific CT aspects while students are building their computational models through the modeling process. Table 1 presents the PBL features and activities to support teachers and students’ CT through modeling.

**Table 1:** PBL design features, PBL strategies/activities, and associated teacher and student supports to CT

PBL design features	PBL key strategies and activities [Supports to CT aspects]
Focus on learning goals	Develop performance-based learning goals by integrating core ideas with science practices that intersect with CT [CT1]
Start with diving questions	Use Driving Question Board [CT1, 5]
Participate in science practices	Select science practices intersected with CT, constructing and revising models, and develop associate learning activities M1. Characterize problem or phenomenon to model ● Use Driving Question Board [CT1] M2. Define the boundaries of the system ● Develop mechanistic model illustration [CT1] ● Create a computational model using SageModeler focusing on **measurable key elements (variables)** [CT2] M3. Design and construct model structure ● Create a computational model using SageModeler focusing on **relationships among variables** [CT2] M4. Test, evaluate, and debug model behavior ● Run simulation [CT3] ● Generate graphs using simulation output [CT4] M5. Use model to explain and predict behavior of phenomenon or design solution to a problem ● Run simulation [CT5] ● Generate graphs using experimental or real-world data [CT4]
Create a set of tangible products through collaborative activities	Design collaborative learning activities for students to create products ● Work in pairs; Share and evaluate products; Communicate their products to others [CT3] Student products ● Mechanistic model illustrations [CT1] ● Computational models [CT2] ● Data representations [CT3, 4] ● Written explanations [CT5]
Scaffold with learning technologies	Select technology tools to engage students in CT and support collaborative learning ● Driving Question Board [CT1, 5] ● SageModeler modeling tool [CT2, 3, 4]

*CT1* Problem decomposition, *CT2* Computational artifacts creation, *CT3* Testing and debugging, *CT4* Generating, organizing, and interpreting data, *CT5* Iterative refinements

#### M1

*To support students in the CT aspect of problem decomposition*, our curriculum used design feature 2, *Driving Questions*, as described above to engage students in identifying essential elements for their models by developing and discussing a driving question and observing various phenomena collaboratively. This activity helps students to characterize a phenomenon by understanding the various parts of the phenomenon and the interconnections between these parts that are necessary for building a model.

#### M2

**Fig. 5:**
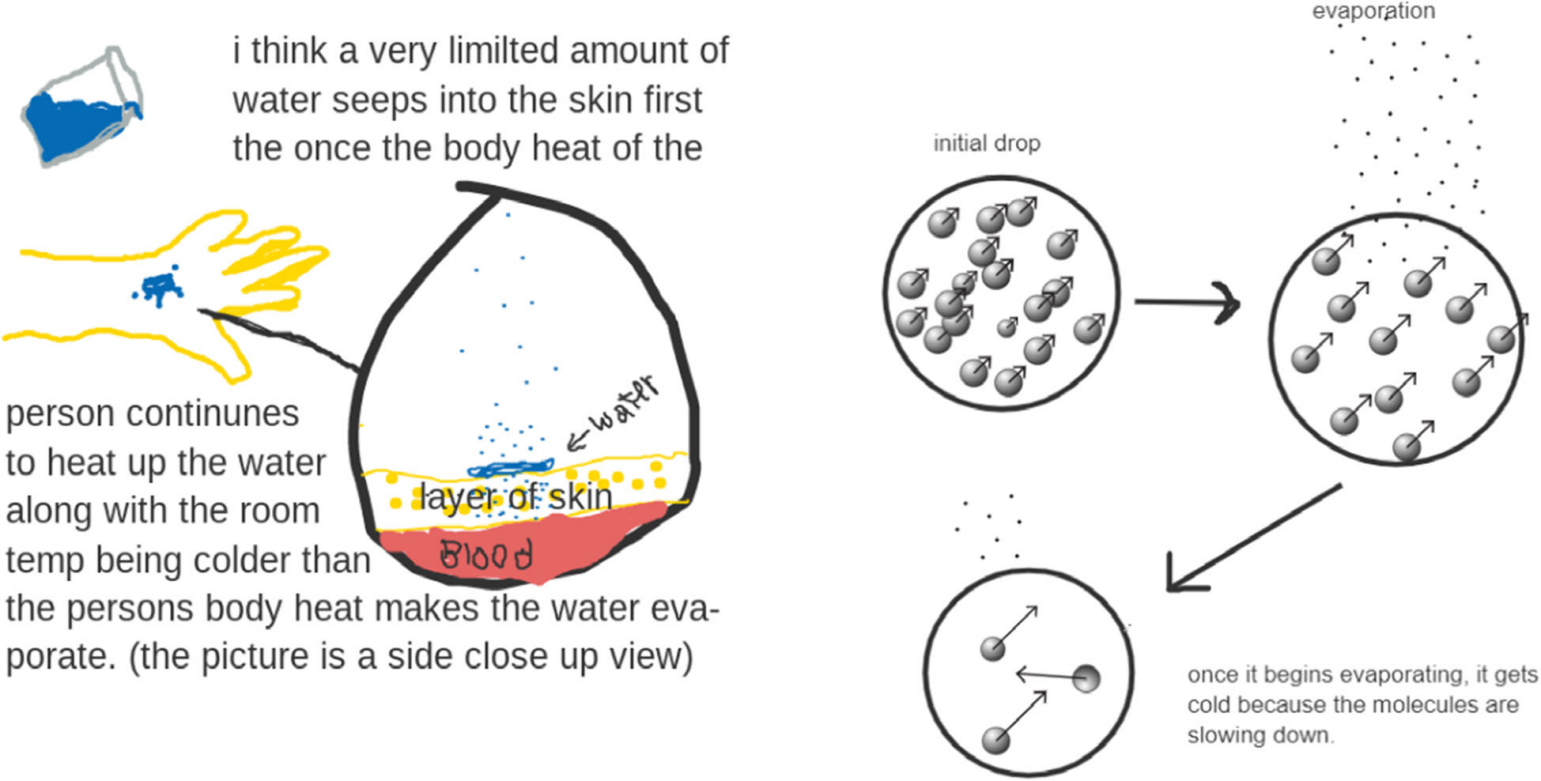
Students’ mechanistic modeling illustration

*To support students in the CT aspects of problem decomposition and creating artifacts* within the *defining boundaries of the system* in the modeling process, our curriculum materials scaffold students in defining the key elements of a phenomenon through a mechanistic “model illustration” (e.g., paper-pencil models). For example, students first experience how different liquids (water, acetone, rubbing alcohol, and cooking oil) evaporate at different rates when placed on their skin. They then describe their observation of the “evaporative cooling” phenomenon through a mechanistic “model illustration” of the phenomenon. In order to construct a “model illustration” students first need to identify elements (e.g., the heat of the skin, the “viscosity” of the liquid) that are important for describing the behavior of the phenomenon. The process of constructing an explanatory “model illustration” of the phenomenon helps students define the phenomenon by encouraging them to consider the phenomenon not as a holistic event when they participate in the driving question and observation phenomenon activities, and but as a series of elements that are interconnected with each other (see Fig. 5).

In a follow-up discussion, students are tasked with breaking down these elements into more specific elements that are directly related to answering the driving question through further investigations. This discussion activity focuses on “setting a boundary of a model” by narrowing down the selection of key elements related to the driving question. For example, a student might draw “heat” in various forms in their model illustrations, but through subsequent discussions, they begin to recognize that heat (thermal energy) is being transferred from their skin to the liquid particles and that as the liquid particles are gaining more thermal energy (and therefore moving faster), they are able to break free from the other particles that make up the liquid and evaporate. The elements are changed from a macro-level phenomenon (e.g., heat and liquid) to a micro-level scale (thermal energy and liquid molecules) to explain the evaporation of liquids. This helps students narrow the scope of the phenomenon by identifying the key elements in the phenomenon that are necessary for explaining the phenomenon in computational models. These activities support students in engaging with “*problem decomposition*” by providing the opportunity to explore important and relevant elements in a phenomenon for specifying the scope of the model (Bielik et al., 2019; Krajcik & Czerniak, 2018).

**Fig. 6:**
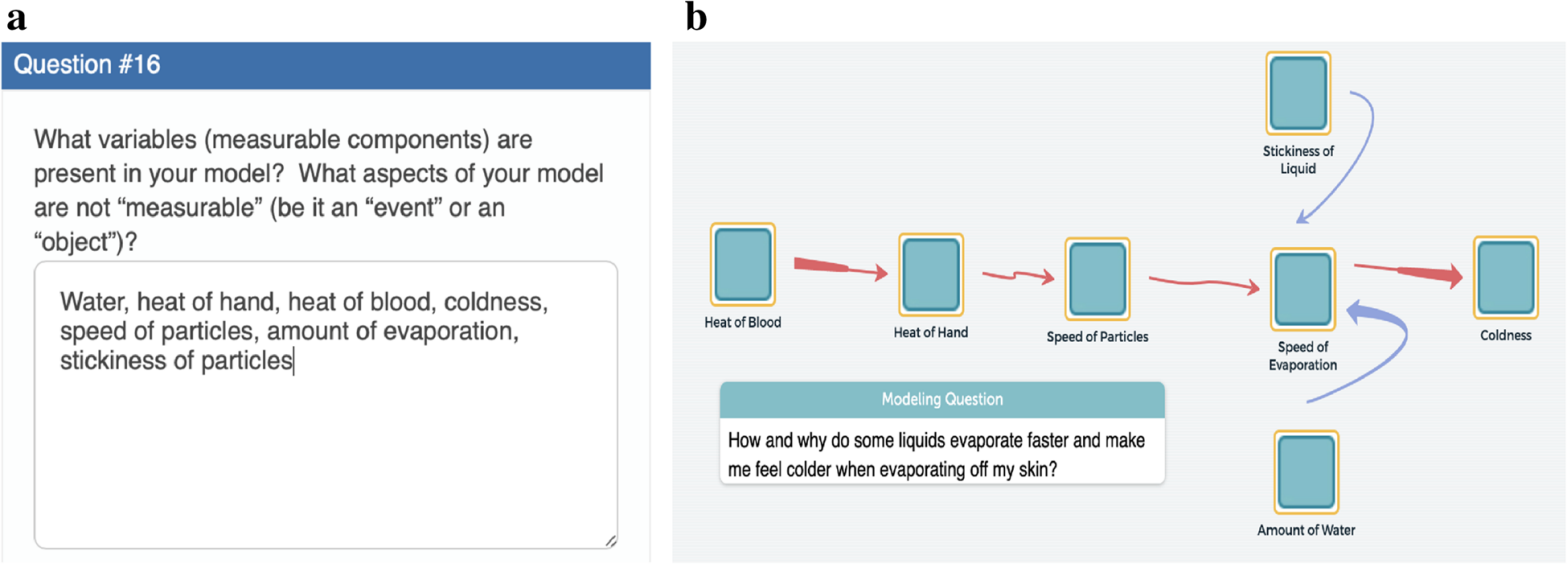
Creating a computational model (artifacts) focusing on elements. **a** Student listing of variables. **b** Encode the variables in SageModeler

Once students have developed their model illustrations, they can proceed to create a computational model focusing on the elements they defined through problem decomposition. For example, T and L (students) identified and listed the elements of the system including water, heat, hand, coldness, and stickiness that they have identified through their discussions of the driving question and their creation of their model illustrations. They then took this list of elements and discussed how they can be reimagined as measurable (or calculatible) variables so that they can be used to create an initial computational model that is computationally runnable (Fig. 6a). Naming the elements to be measurable is important for engaging students in CT to find a computational solution because having computationally compatible elements is vital for the development of computational models that can be simulated. The students finally added their newly identified variables into the computational modeling software, called SageModeler as shown in Fig. 6b.

#### M3

**Fig. 7:**
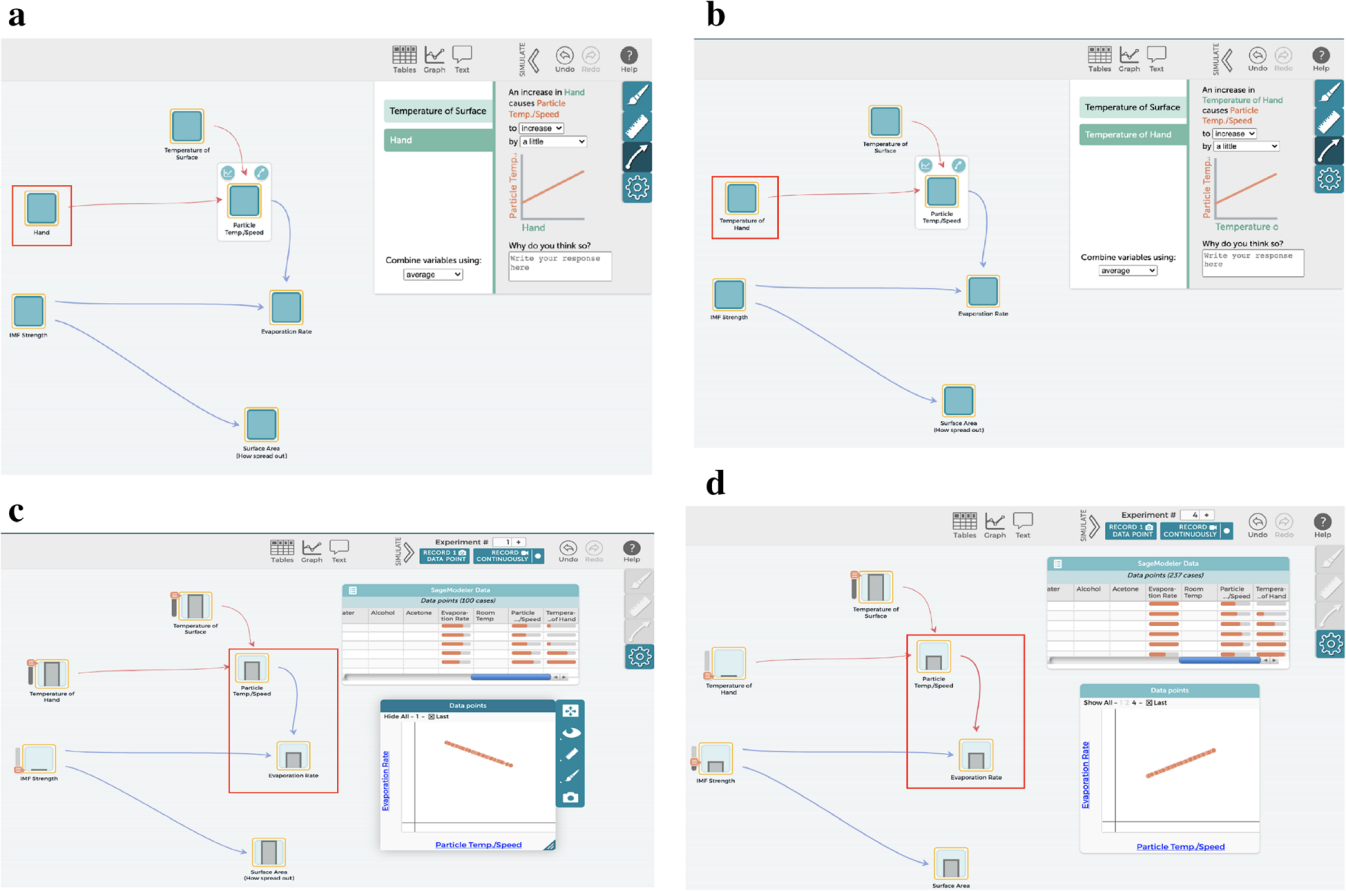
Creating a computational model (creating artifacts) focusing on relationships among variables. **a** Building Relationship between variables. **b** Revise the name of elements to measurable variables. **c** Generation graphs through simulation. **d** Revise the relationships based on the generated graph

*To support students in the CT aspect of creating artifacts* in the *design and construction of model structure* of the modeling process, our PBL curriculum provides various activities that focus on understanding the relationships among elements to explore those relationships to produce the outputs that students observed in the phenomenon. Some activities that allow for students to explore the causal relationships between variables are: classroom experiments where students collect qualitative and/or quantitative data, computerized simulations where students explore various aspects of the phenomenon in a virtual world, and classroom discussions built around creating explanations for how two or more aspects of the phenomenon interact. After exploring the causal relationships between variables, students need to add them into their computational models. Setting computational relationships between variables is important for providing students with opportunities to partake in CT through algorithmic thinking and for creating computational models that can be recognized and simulated by a computer. For example, after selecting essential elements, Q and R (students) defined the input and output variables of the system and specified the relationships between variables so that the model can be simulated as they intended. When they chose a relationship, the students were asked to make sure the sentence is meaningful and matches their conceptual understanding of the phenomenon (see Fig. 7a). Particularly, the name of the elements should be measurable so that the computer can reliably simulate the relationship between these variables, which is vital for engaging students in CT. For example, Q and R read the sentence shown in the relationship window, “An increase in Hand causes Particle Temp./Speed to increase by a little” (Fig. 7a see a red line box) and quickly recognized that the “Hand” itself does not increase. So they changed the “Hand” to be a measurable variable called “temperature of Hand” to reflect their conceptual understanding that the temperature of the hand was causing the temperature of the liquid particles to increase (Fig. 7b). After setting the relationships between the variables of their model, Q and R ran simulations and then scrutinized those relationships by using the relationship testing features embedded in SageModeler.

#### M4

*To support students in the CT aspects of organizing and interpreting data, and testing and debugging* in the *testing, evaluating, and debugging model behaviors* of the modeling process, students run a simulation to test and revise their computational models. During these simulations, students manipulate the initial values of input variables to explore the effects of those changes on the output variables in a whole system. Using simulation output students can generate graphs to examine the patterns of the relationships among variables detecting issues in an inappropriate solution. When the outputs of their simulation do not match those of their experimental data or conceptual understanding, students rework their models and confirm the solution using multiple starting conditions. In particular Q and R (students) used the “simulate” feature of the program to generate output from their model that they displayed as a graph (Fig. 7c). As this graph of “Particle Temp./Speed” vs. “Evaporation Rate” showed a negative trendline and therefore did not match their conceptual understanding of the phenomenon. The students then identified the source of the inappropriate trends between Particle Temp./Speed and Evaporation Rate and revised the relationship appropriately (Fig. 7d).

#### M5

**Fig. 8:**
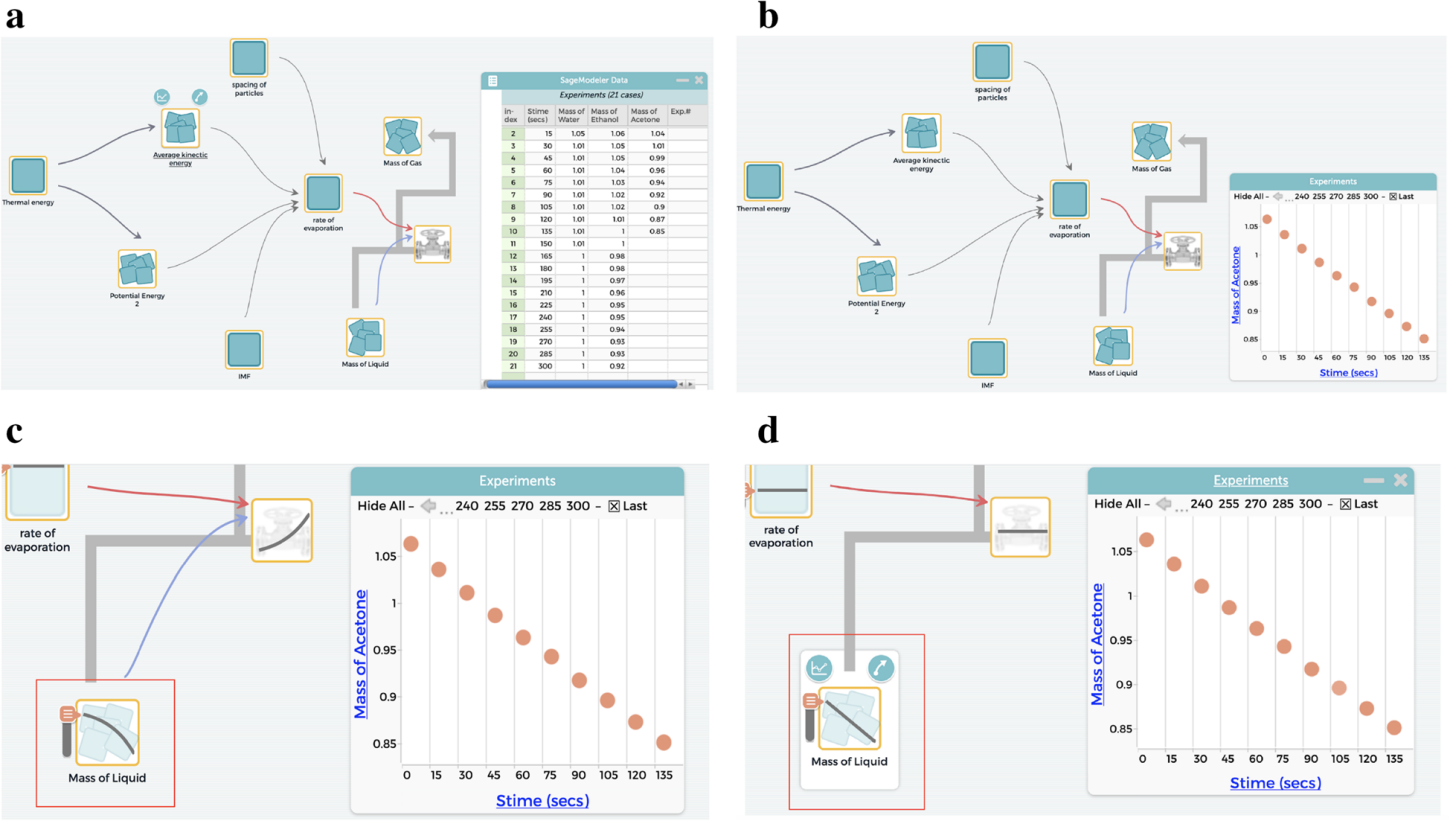
Using models to predict novel phenomena and to validate models with experimental data for making iterative refinements. **a** Model with experimental data. **b** Graph from experimental data. **c** Graph from simulated output (in red box). **d** Revised Model with simulated graph (in red box)

*To support students in the CT aspect of organizing and interpreting data, and making iterative refinements* in the *using models to explain the behavior of a phenomenon* of the modeling process***,*** learning activities are designed for students to compare their simulation generated output to experimental and/or real-world data by generating graphs for validating their models. For example T and A (students) collected data on how the mass of each of the three main liquids (ethanol, acetone, and water) decreased during the process of evaporation and added this data into SageModeler (Fig. 8a). The students then used SageModeler to generate a graph of this real-world data, which showed that the mass of acetone decreased at a constant rate during this experiment (Fig. 8b). T and A then used the “simulate” feature to generate output from their model, which showed that the mass of the liquid (in this case Acetone) decreased by “less and less” during the process of evaporation (Fig. 8c). As this simulation generated output did not match their externally generated experimental data, T and A made adjustments to their model. They removed the connection between the mass of the liquid and the evaporation rate of the liquid (as measured by the valve on the transfer link between the “Mass of liquid” and “mass of gas” variables). This made the rate of evaporation a constant and therefore allowed the “mass of liquid” to decrease at a constant rate, allowing the model to match the experimental data (Fig. 8d).

Students engage in CT through refining their models iteratively using real-world data to explain and predict novel phenomena. When students use their model to anticipate the outcome of novel phenomena, they examine the usability of their model by reading and interpreting the model as a series of interconnected relationships between variables and making sense of the data output of the model. Through these PBL activities, students are able to articulate the differences between their model and the underlying phenomenon, reflecting on both the limitations and usability of their model.

### Design feature 4. Creating a set of tangible products through collaborative activities

PBL focuses on product development for actively applying science ideas and engaging in science practices through building, refining and reflecting on their products. Creating products supports students in developing a concrete and explicit representation of their emerging knowledge (National Academies of Sciences, Engineering, and Medicine, 2018) related to explaining a phenomenon. Similarly, collaborative activities help students build shared understandings of scientific ideas and of the nature of the discipline as they engage in discourse with their classmates and with teachers (Brown & Campione, 1994).

**Fig. 9:**
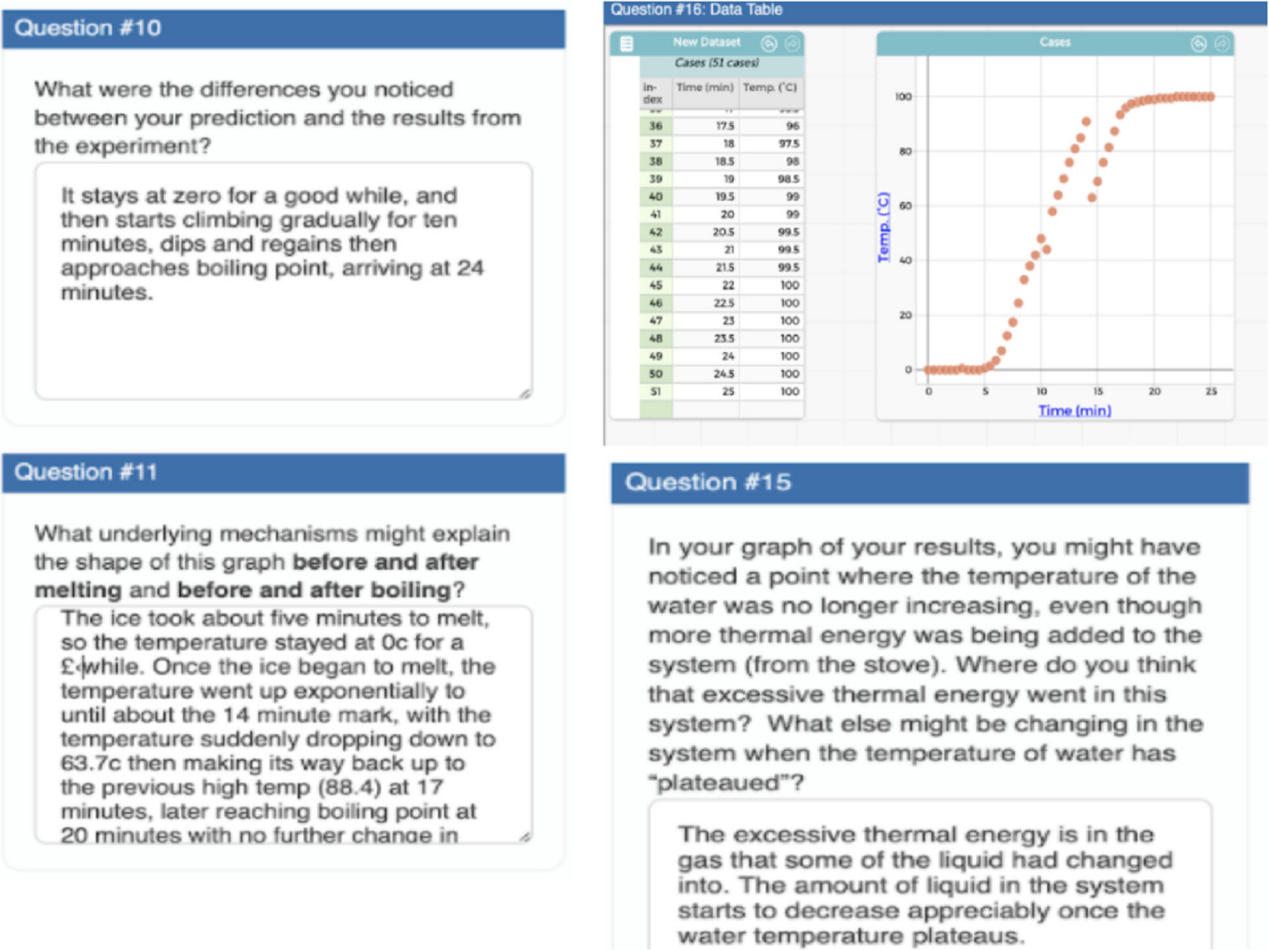
Student written responses of generation and interpretation of data

In our curriculum materials, the tangible products are the mechanistic model illustrations (Fig. 5), computational models (Fig. 1), data representations (Fig. 8), and written explanations that result from students applying some CT aspects (Fig. 9), if not all, in an iterative manner to answer a driving question. Throughout the curriculum development, we designed our materials as collaborative learning environments that allow students to continuously build and revise these artifacts throughout the unit. For example, students work in pairs to build and revise their models of the phenomenon of evaporative cooling. They also work collaboratively to gather evidence (through analyzing experimental data of a “heating curve” of water) to support their models (Fig. 9).

Within this unit, teachers also regularly facilitate whole-class discussions (often focused on analyzing experimental data) that foster collaborative sensemaking. Students are also given frequent opportunities to share their models with their peers and receive constructive feedback, thereby engaging with the CT aspect of *testing and debugging*. To help teachers and students give valuable feedback to students or peers, we provided them with written descriptions of evaluation criteria for reviewing models and giving feedback. Our criteria for model evaluation includes: 1) the model uses appropriately named variables, 2) the model appropriately defines relationships between these variables, 3) the model has clearly defined system boundaries, and 4) the model accurately simulates the behavior of the system. After completing their models, students communicate their solutions including the results of their data analysis to others. This can be done via an oral presentation or through written explanations. This design feature supports the CT aspects of *creating computational artifacts through testing and debugging*, and *generating and interpreting data*. While students develop those artifacts collaboratively, students engage in CT through 1) decomposing a problem (or phenomena) by breaking it into essential elements, 2) encoding logically and algorithmically those elements in computational models so that they can be runnable or usable through simulation, 3) generating graphs using experimental/real-world data and simulated output to validate the computational models, 4) testing and debugging issues by comparing the results of generation and simulation output with experimental and real-world data along with utilizing feedback from peers and teachers, and 5) making iterative refinements.

### Design feature 5. Scaffolding with learning technologies

We select technology tools with the consideration of two criteria: (1) do they engage students in science practices through active construction of tangible knowledge products, and (2) do they support collaborative learning environments for construction, feedback, reflection, and revisions of artifacts. We primarily used two technology tools in this unit: Driving Question Boards (DQBs) (see Fig. 3) and a modeling tool, called SageModeler (see Fig. 1). Driving Question Boards are collaborative tools that enable students to share ideas with classmates or group members. DQBs allow for students to see questions that have been posted by their classmates, rearrange these questions into meaningful categories, and anonymously post possible answers to these questions as the unit progresses. DQBs can take many forms including physical whiteboards with sticky notes, virtual whiteboards (such as Jamboards), or shared Google Docs Document. The DQB supported students in engaging with the CT aspects of “*problem decomposition*” and “*iterative refinement*” by providing space for students to revisit different aspects of the phenomenon that they had previously ignored and therefore encouraging them to make additional revisions to their models. When students obtained new information and new data, they revisited the DQB to answer questions they previously had, revise earlier answers based on new evidence, and ask new questions. Working with the DQB encourages students in the process of revising their answers and models, which is key for iterative refinement of the model to answer the driving question.

SageModeler (https://sagemodeler.concord.org) is a free, web-based, open-source tool to scaffold student learning so that young students, beginning in middle school, can engage in computational thinking through building, testing, sharing, commenting, evaluating, and revising models. The characteristics of SageModeler that support students in computational thinking while respecting learner variability include 1) visual and textual representations of variables and relationships that are customizable by the learner, 2) a simple drag-and-drop interface for constructing a model, 3) the ability to define functional relationships between variables semi-quantitatively without having to write equations, 4) simulation and sharing functions for testing, debugging, and commenting on a model, and 5) multiple pathways for generating visualizations of model output.

Defining relationships qualitatively helps students overcome the mathematical obstacles typically associated with creating computational models, and allows them to focus on their conceptual understandings. The goal of this design feature is to scaffold the CT aspect of *creating artifacts using algorithmic thinking* for a diverse range of students. In SageModeler, students build computational models without coding or programming. Rather, in SageModeler students add input, output, and intermediate elements, and set the relationship among those elements using words (Fig. 7). When students set these relationships, they need algorithmic thinking to precisely define the causal relationships among these elements so that the computer can run a simulation to predict phenomena. The model simulations make it possible for students to see relationships among elements (e.g. how the relationships in a set of sub-elements affect the model behavior as a whole) by manipulating initial values or changing the relationship among elements. Additionally, students can make use of an embedded exploratory data analysis environment designed for students. To better understand and improve their models, students can compare the output of their model with an experimental or real-world data set. These functions support CT in *iterative refinement* through the *generation of data* and *testing, debugging, and revising*.

## Conclusion and future direction

Computational thinking (CT) has gained tremendous attention as a critical competence in our global society to fully explain and predict everyday phenomena or solve complex problems that require computational solutions. Although there is a growing need for CT and there have been efforts to develop CT curricular materials in K-12 education (Grover & Pea, 2017; Meadows, 2008), it has yet to achieve promising success in schools. The key challenges for STEM educators are how to a) integrate CT seamlessly into multiple STEM courses, and b) engage students in CT by providing effective support.

In order to meet such needs, we propose PBL that uses computational modeling as a way to engage CT. In PBL, students engage in compelling, complex, and meaningful problems that are important to them and the activities learners perform mirror what scientists do. Furthermore, computational modeling provides opportunity for students to apply their knowledge as well as engage in all aspects of CT for producing a wide range of tangible computational products (e.g., computational simulation models, generation of graphs using simulation output and real-world data) through building, testing, evaluating, and revising models. Such a PBL science classroom supports students to explore phenomena, investigate questions, discuss their ideas, engage in CT, challenge the ideas of others, try out new ideas, and construct and revise computational models.

In this paper, we illustrate how the PBL design features can support students in engaging with CT through modeling. We believe our curriculum development efforts extend the learning of CT in K-12 education by providing a principle based approach for developing technological, curricular, and pedagogical supports to engage students in CT through modeling. Our design features are developed by incorporating the critical aspects of three fields proposed by scholars in CT (Grover & Pea, 2017; Weintrop et al., 2016; Wing, 2006), modeling (Schwarz et al., 2017), and PBL (Krajcik & Shin, in press). Our curriculum presents how these theories can be applied practically in a systematic way for developing learning and teaching materials. Since these designs have become progressively better through a process of iterative design experiments, we are in the process of developing science curricula in biology, earth science, and physics, and implementing them in various contexts of high school classrooms in science. As such, the PBL in a context of modeling is a principled approach for supporting CT across the curriculum. The PBL features have promising benefits for promoting science learning and CT for all students including students who historically have not had access to STEM careers. PBL can equip all students with the intellectual capabilities of CT necessary to understand phenomena around their world and solve problems computationally.

## Data Availability

Data sharing is not applicable to this article as no datasets were generated or analysed during the current study.

## Footnotes

1 Big ideas of science are aligned with disciplinary core ideas and crosscutting concepts in A Framework for K-12 Science Education (National Research Council (NRC), 2012) and U.S. Next Generation Science Standards (NGSS Lead States, 2013).
